# Haplotypes of *P2RX7* gene polymorphisms are associated with both cold pain sensitivity and analgesic effect of fentanyl

**DOI:** 10.1186/1744-8069-10-75

**Published:** 2014-12-03

**Authors:** Soichiro Ide, Daisuke Nishizawa, Ken-ichi Fukuda, Shinya Kasai, Junko Hasegawa, Masakazu Hayashida, Masabumi Minami, Kazutaka Ikeda

**Affiliations:** Addictive Substance Project, Tokyo Metropolitan Institute of Medical Science, 2-1-6 Kamikitazawa, Setagaya-ku, Tokyo, 156-8506 Japan; Department of Pharmacology, Graduate School of Pharmaceutical Sciences, Hokkaido University, Sapporo, Japan; Department of Dental Anesthesiology, Tokyo Dental College, Tokyo, Japan; Department of Anesthesiology & Pain Medicine, Juntendo University School of Medicine, Tokyo, Japan

**Keywords:** P2X_7_ receptor, ATP, Purinergic receptor, Single-nucleotide polymorphism, Pain, Fentanyl, Cold pain, Haplotype analysis, Perioperative analgesia

## Abstract

**Background:**

The P2X_7_ receptor is a member of the P2X family of adenosine 5′-triphosphate-gated cation channels. Several recent studies have demonstrated that this receptor is involved in mechanisms related to pain and inflammation. However, unknown is whether polymorphisms of the *P2RX7* gene that encodes the human P2X_7_ receptor influence pain sensitivity and analgesic effects of opioids. The *P2RX7* gene is known to be highly polymorphic. Thus, the present study examined associations between fentanyl sensitivity and polymorphisms in the *P2RX7* gene in 355 Japanese patients who underwent painful orofacial cosmetic surgery.

**Results:**

We first conducted linkage disequilibrium (LD) analyses for 55 reported single-nucleotide polymorphisms (SNPs) in the region within and around the *P2RX7* gene using genomic samples from 100 patients. In our samples, 42 SNPs were polymorphic, and a total of five LD blocks with six Tag SNPs (rs2708092, rs1180012, rs1718125, rs208293, rs1718136, and rs7132846) were observed. Thus, we further analyzed associations between genotypes/haplotypes of these Tag SNPs and clinical data using a total of 355 samples. In the genotype-based association study, only the rs1718125 G > A SNP tended to be associated with higher pain scores on a visual analog scale 24 h after surgery (VAS24). The haplotype-based association study showed that subjects with homozygous haplotype No.3 (GTAAAC; estimated frequency: 15.0%) exhibited significantly higher cold pain sensitivity and lower analgesic effects of fentanyl for acute cold pain in the cold pressor test. Conversely, subjects who carried haplotype No.1 (ACGGAC; estimated frequency: 24.5%) tended to exhibit lower cold pain sensitivity and higher analgesic effects of fentanyl. Furthermore, subjects with homozygous haplotype No.2 (GCGGAC; estimated frequency: 22.9%) exhibited significantly lower VAS24 scores.

**Conclusions:**

Cold pain sensitivity and analgesic effects of fentanyl were related to the SNP and haplotypes of the *P2RX7* gene. The patients with the rs1718125 G>A SNP tended to show higher VAS24 scores. Moreover, the combination of polymorphisms from the 5′-flanking region to exon 5 recessively affected cold pain sensitivity and analgesic effects of opioids for acute cold pain. The present findings shed light on the involvement of *P2RX7* gene polymorphisms in naive cold pain sensitivity and analgesic effects of fentanyl.

**Electronic supplementary material:**

The online version of this article (doi:10.1186/1744-8069-10-75) contains supplementary material, which is available to authorized users.

## Introduction

Extracellular adenosine 5′-triphosphate (ATP) has been recognized as a neurotransmitter and/or neuromodulater in the nervous system that specifically acts on P2 purinergic receptors on the cell surface. P2 purinergic receptors are divided into two classes. P2X receptors are ATP-gated cation channels and subdivided into seven subtypes (P2X_1–7_). P2Y receptors are heptahelical G-protein-coupled receptors and subdivided into eight subtypes (P2Y_1_, P2Y_2_, P2Y_4_, P2Y_6_, P2Y_11_, P2Y_12_, P2Y_13_, and P2Y_14_). Both P2X and P2Y receptors are widely expressed in the sensory nerve system and exert various effects on neuronal and glial cells [1]. Recent studies revealed that ATP and its receptors are involved in peripheral and central nociceptive transmission, including mechanisms involved in neuropathic pain [2, 3].

P2X_7_ receptors exhibit unique pharmacological characteristics compared with other P2X receptor subtypes. A high concentration of ATP (i.e., >100 μM) is required for P2X_7_ receptor activation [4]. In addition to acting as ATP-gated Ca^2+^-permeable cation channels, P2X_7_ receptors induce the formation of large nonselective pores with a 900 Da cut-off [4, 5]. Many studies have shown the involvement of P2X_7_ receptors in pain. P2X_7_ receptor knockout mice have been shown to exhibit a reduction of thermal and mechanical hypersensitivity in a partial sciatic nerve ligation model [6]. Recent developments in selective inhibitors of P2X_7_ receptors also showed that P2X_7_ receptor blockade reduced nociceptive behavior in several animal models of neuropathic and inflammatory pain [7–10]. Although these studies revealed an important role for P2X_7_ receptors in neuropathic and inflammatory pain development in animal models, the involvement of P2X_7_ receptors in the modulation of naive pain sensitivity and efficacy of analgesics in humans is still unclear.

Opioid analgesics, such as fentanyl and morphine, are widely used for the treatment of moderate to severe pain. However, the analgesic efficacy of opioids is well known to vary widely among individuals [11]. Individual differences may be related to various genetic and nongenetic factors, including gender, age, ethnic origin, hepatic or renal function, and mental status [12]. Several studies that used mice that lack the μ-opioid receptor (MOP) [13–15] have shown that analgesia produced by opioids crucially depends on the level of MOP expression. Furthermore, several single-nucleotide polymorphisms (SNPs) in the *OPRM1* gene, which encodes the human MOP protein, have been reported to lead to differences in the analgesic efficacy of opioids [16]. Several gene-association studies have also reported that the analgesic efficacy of opioids could be affected by other molecules [17–21].

Many gene polymorphisms, most of which are SNPs, reportedly exist in the genes that encode P2X and P2Y receptors. The gene that encodes the human P2X_7_ receptor (*P2RX7*) is known to be highly polymorphic. Some SNPs in the *P2RX7* gene have been shown to cause changes in receptor function [22–24]. Only a few studies have tested associations with human pain sensitivity [24], and whether genetic polymorphisms in the *P2RX7* gene exhibit associations with pain sensitivity or opioid analgesia is still unclear. In contrast to animal studies that use standardized pain tests, the analgesic effects of opioids in humans are usually evaluated in patients with actual pain, particularly cancer pain or acute postoperative pain [16]. Patients with acute postoperative pain following standardized surgical procedures may be more optimal subjects for investigating gene-opioid effect relationships [11, 17, 25]. Furthermore, because subjects prior to cosmetic orthognathic surgery have no spontaneous pain, the analgesic effects of opioids in humans can be evaluated under more optimal conditions. Therefore, the present study examined whether SNPs and haplotypes in the *P2RX7* gene affect cold pain sensitivity and the analgesic effects of fentanyl, one of the most commonly used opioid analgesics, evaluated by a standardized pain test and fentanyl requirements in healthy Japanese subjects who underwent uniform surgical procedures.

## Materials and methods

### Ethics statement

The study protocol was approved by the Institutional Review Board, Tokyo Dental College, Chiba, Japan, and the Institutional Review Board, Tokyo Metropolitan Institute of Medical Science, Tokyo, Japan. Written informed consent was obtained from all of the patients and also from parents if required.

### Patients

Enrolled in the study were 355 healthy patients (American Society of Anesthesiologists Physical Status I, age 15–52 years, 125 males and 230 females [the same patients who served as subjects in our previous report] [17]) who were scheduled to undergo cosmetic orthognathic surgery (mandibular sagittal split ramus osteotomy) for mandibular prognathism at Tokyo Dental College Suidoubashi Hospital. Patients with chronic pain, those taking pain medication, and those who had experienced Raynaud’s phenomenon were excluded.

### Preoperative cold pressor-induced pain test

The patients were premedicated with oral diazepam, 5 mg, and oral famotidine, 150 mg, 90 min before the induction of anesthesia. The patients had an intravenous (i.v.) line on the forearm on their nondominant side. The temperature in the operating room was maintained at 26°C. The cold pressor-induced pain test was then performed before and 3 min after an i.v. bolus injection of fentanyl, 2 μg/kg, as previously described [25, 26]. Briefly, crushed ice cubes and cold water were blended 15 min before the test in a 1 L isolated tank, and the mixture was stirred immediately before each test to ensure uniform temperature distribution (0°C) within the tank. The dominant hand was immersed up to the wrist. Patients were instructed to keep the hand calm in the ice-cold water and withdraw it as soon as they perceived any pain. All of the patients were administered the test by the same investigator. The baseline latency to pain perception, defined as the time of immersion of the hand in the ice water, before an i.v. injection of fentanyl (PPLpre) was recorded. A cut-off point of 150 s was set to avoid tissue damage. The hand was warmed with a hair dryer as soon as it was withdrawn from the ice water until the sensation of cold was completely abolished. The patients then received i.v. fentanyl, 2 μg/kg. Three minutes after the injection, the pain perception latency of the dominant hand (PPLpost) was measured again. The analgesic effect of fentanyl in the preoperative cold pressor-induced pain test was evaluated simply as the difference between PPLpost and PPLpre (PPLpost - PPLpre).

### Anesthesia, surgery, and postoperative pain management

Anesthesia, surgery, and postoperative pain management were performed as previously described [17]. Briefly, after the cold pressor-induced pain test ended, general anesthesia was induced with a target-controlled infusion (TCI) of propofol. After the induction of anesthesia, 10 ml of venous blood was sampled for the preparation of DNA specimens. Local anesthesia was performed on the right side of the surgical field with 8 ml of 2% lidocaine that contained epinephrine, 12.5 μg/ml, and right mandibular ramus osteotomy was performed. Local anesthesia was then performed on the left side, and left mandibular ramus osteotomy was performed. The bilateral mandibular bone segments were fixed in appropriate positions. Whenever systolic blood pressure or heart rate exceeded +20% of the preinduction value during surgery, i.v. fentanyl, 1 μg/kg, was administered. At the end of the surgery, rectal diclofenac sodium, 50 mg, and i.v. dexamethasone, 8 mg, were administered at the request of surgeons to prevent postoperative orofacial edema/swelling. After emergence from anesthesia and tracheal extubation, i.v. patient-controlled analgesia (PCA) with a fentanyl-droperidol combination (2 mg fentanyl and 5 mg droperidol diluted in normal saline in a total volume of 50 ml) commenced using a CADD-Legacy PCA pump (Smiths Medical Japan, Tokyo, Japan). A bolus dose of fentanyl, 20 μg, on demand and a lockout time of 10 min were set. Patient-controlled analgesia continued for 24 h postoperatively. The intensity of spontaneous pain was assessed 3 and 24 h postoperatively using a 100-mm visual analog scale (VAS), with 0 mm indicating no pain and 100 mm indicating the worst pain imaginable. Intraoperative fentanyl use and postoperative PCA fentanyl use during the first 24 h postoperative period were recorded. Doses of fentanyl administered intraoperatively and postoperatively were normalized to body weight. Additionally, perioperative fentanyl use was calculated as the sum of intraoperative fentanyl use and postoperative fentanyl use.

### Genotyping procedures and linkage disequilibrium analysis

Genomic DNA was extracted from whole-blood samples using standard procedures. The extracted DNA was dissolved in TE buffer (10 mM Tris–HCl, 1 mM ethylenediaminetetraacetic acid, pH 8.0). The DNA concentration was adjusted to 5–50 ng/μl for genotyping individual SNPs or 100 ng/μl for whole-genome genotyping using a NanoDrop ND-1000 Spectrophotometer (NanoDrop Technologies, Wilmington, DE, USA).

For the analysis of SNPs within and around the *P2RX7* gene region, genotype data from whole-genome genotyping were used. Briefly, whole-genome genotyping was performed using Infinium assay II and an iScan system (Illumina, San Diego, CA) according to the manufacturer’s instructions. Five kinds of BeadChips were used to genotype 40, 67, 6, 119, and 123 samples, respectively: HumanHap300 (total markers: 317,503), HumanHap300-Duo (total markers: 318,237), Human610-Quad v1 (total markers: 620,901), Human1M v1.0 (total markers: 1,072,820), and Human 1 M-Duo v3 (total markers: 1,199,187). Some BeadChips included a number of probes specific to copy number variation markers, but most were for SNP markers on the human autosome or sex chromosome. Approximately 300,000 SNP markers were commonly included in all of the BeadChips. After the whole-genome genotyping, the data for genotyped samples were analyzed using BeadStudio or GenomeStudio with the Genotyping module v3.3.7 (Illumina) to evaluate the quality of the results, and the genotype data for all of the SNPs with *P2RX7* gene annotation were extracted. In the data-cleaning process, markers that had “Cluster sep” values (i.e., an index of genotype cluster separation) <0.4 and were separated from any of the three genotype clusters were excluded from the subsequent association study.

Single-nucleotide polymorphisms for the association studies were selected based on recently advanced tagging strategies [27–29]. To identify relationships between the SNPs used in the study, linkage disequilibrium (LD) analysis was performed in 55 SNPs that were in the approximately 108 kbp region that contained the *P2RX7* gene among 1,072,820 markers in the Human 1 M v1.0 BeadChip for 100 samples using Haploview v.4.2 [30]. For the estimation of LD strength between the SNPs, the commonly used *D’* and *r*^*2*^ values were pairwise calculated using the genotype dataset of each SNP. Linkage disequilibrium blocks were defined among the SNPs that showed “strong LD,” based on the default algorithm of Gabriel et al. [31], in which the upper and lower 95% confidence limits on *D’* for strong LD were set at 0.98 and 0.7, respectively. Tag SNPs in the LD block were consequently determined using Tagger software with default settings, which is incorporated in Haploview and has been detailed in a previous report [29].

### Statistical analysis

Parametric and nonparametric data are expressed as mean ± SD and median [interquartile range], respectively. The statistical analysis was performed using IBM SPSS statistics v.20.0.0 (IBM, Tokyo, Japan). In the present study, none of the clinically measured endpoints that were related to pain sensitivity (i.e., PPLpre) or fentanyl analgesia (i.e., analgesia measured with the preoperative cold pressor test, perioperative fentanyl use, and VAS scores at 3 and 24 h postoperatively) were normally distributed. Therefore, nonparametric analyses, including the Mann–Whitney *U*-test or Kruskal-Wallis test (with Steel-Dwass multiple comparison tests), were used to detect possible associations between any of the clinical or genomic parameters (e.g., sex and genotypes of the Tag SNP) and clinical endpoints related to pain sensitivity or the analgesic effects of fentanyl. The sample size of the present nonparametric data was higher than the estimated size that possesses statistical power (1 minus type II error probability) of 99% for the Cohen’s conventional “medium” effect size of 0.25, when power analysis was performed for analysis of variance with three genotype groups using G*Power v.3.1.3 [32]. Haplotype analyses were performed using HPlus v.3.2 software with default settings (Fred Hutchinson Cancer Research Center, Seattle, WA, USA) that employs expectation-maximization with a modified progressive ligation computational algorithm to infer haplotypes [33].

## Results

To identify the LD blocks in the approximately 108 kbp region that contains the *P2RX7* gene, 55 SNPs among 1,072,820 markers that were included in the whole-genome genotyping (Human 1 M v1.0 BeadChip) were tested using genomic samples of 100 patients who were randomly selected from all 355 Japanese patients. In these Japanese samples, 42 SNPs were found to be polymorphic. A total of five LD blocks (LD1-5) were observed within and around the *P2RX7* gene region (the exon, intron, and approximately 45 kbp 5′-flanking region and 10 kbp 3′-flanking region of the *P2RX7* gene [approximately 53 kbp]), and six representative Tag SNPs (rs2708092, rs1180012, rs1718125, rs208293, rs1718136, and rs7132846) were selected in this region (Table 1, Additional file 1). Thus, we further examined associations between clinical endpoints and these six representative Tag SNPs using genomic samples from all 355 Japanese patients.

**Table 1:** **Allelic frequencies of SNPs in five LD blocks around the** ***P2RX7*** **gene of Japanese subjects**

rs number of SNPs	Position		Major allele	Minor allele	Frequency	LD block
rs12819523	Flanking_5UTR	-45261	G	A	7.5%	
rs12819930	Flanking_5UTR	-45087	G	A	10.7%	1
rs10492051	Flanking_5UTR	-45012	G	-	0.0%	
*rs2708092	Flanking_5UTR	-44792	A	G	49.0%	1
*rs1180012	Flanking_5UTR	-40230	C	T	38.8%	1
rs2516210	Flanking_5UTR	-35256	T	C	15.9%	1
rs1796421	Flanking_5UTR	-30332	T	C	15.9%	1
rs1796412	Flanking_5UTR	-28966	T	C	18.2%	1
rs7298521	Flanking_5UTR	-28847	C	T	22.9%	1
rs1794899	Flanking_5UTR	-27705	G	A	18.0%	1
rs1796415	Flanking_5UTR	-27667	T	-	0.0%	
rs3892756	Flanking_5UTR	-16010	T	-	0.0%	
rs10849846	Flanking_5UTR	-12255	T	C	21.8%	1
rs208277	Flanking_5UTR	-10097	A	G	41.1%	1
rs12314721	Flanking_5UTR	-4921	C	T	14.9%	1
rs9805004	Flanking_5UTR	-4808	C	T	18.2%	1
rs670541	Flanking_5UTR	-1044	C	T	8.9%	1
rs591874	Intron1	IVS1 + 567	A	C	40.9%	1
rs7959194	Intron1	IVS1 + 5089	G	-	0.0%	
rs11065450	Intron1	IVS1 + 8759	C	A	28.7%	
rs568531	Intron1	IVS1 + 8775	C	T	7.5%	2
rs607094	Intron1	IVS1 + 9566	T	C	6.4%	2
rs208286	Intron1	IVS1-5688	G	-	0.0%	
rs17525809	Exon2	T227C[V76A]	T	C	8.9%	
*rs1718125	Intron2	IVS2 + 263	G	A	28.3%	3
rs10849851	Intron3	IVS3-2061	A	G	21.0%	3
rs1653583	Intron3	IVS3-53	C	T	7.2%	3
*rs208293	Intron4	IVS4-47	G	A	41.1%	3
rs208294	Exon5	T463C[Y155H]	T	C	43.5%	3
rs1186055	Intron5	IVS5 + 206	G	T	39.5%	
rs208296	Intron5	IVS5 + 630	C	T	36.1%	
rs11065464	Intron5	IVS5-1025	C	A	24.3%	
rs208298	Intron5	IVS5-922	G	A	16.6%	4
rs654856	Intron5	IVS5-38	C	A	13.4%	4
rs2857589	Intron7	IVS7 + 486	C	A	5.1%	4
rs503720	Intron7	IVS7-217	G	A	16.1%	4
rs16950860	Exon8	C808T[R270C]	C	-	0.0%	
rs7958311	Exon8	G809A[R270H]	G	A	35.6%	
rs7958316	Exon8	G827A[R276H]	G	-	0.0%	
*rs1718136	Intron8	IVS8-3583	A	G	15.0%	5
rs7137542	Intron8	IVS8-545	G	-	0.0%	
*rs7132846	Intron9	IVS9-16	C	T	12.9%	5
rs1718119	Exon11	G1040A[A348T]	G	A	17.5%	5
rs1653598	Intron11	IVS11 + 34	A	G	14.9%	5
rs2567998	Intron11	IVS11-16	A	-	0.0%	
rs10160951	Exon12	C1289G[P430R]	C	-	0.0%	
rs12829218	Intron12	IVS12 + 133	A	-	0.0%	
rs891781	Intron12	IVS12 + 180	C	T	17.5%	5
rs7312642	Intron12	IVS12-1921	G	T	12.1%	
rs12815078	Intron12	IVS12-101	A	G	6.1%	
rs2230912	Exon13	A1379G[Q460R]	A	-	0.0%	
rs3751144	Exon13	C1422T	C	T	12.9%	
rs3751143	Exon13	A1487C[E496A]	A	C	32.0%	
rs1718161	Flanking_3UTR	+3582	A	G	6.5%	
rs2686365	Flanking_3UTR	+9907	C	-	0.0%	

*Tag SNPs in the LD blocks (selected using Tagger software with default settings; *r* ^*2*^ > 0.8).

Of the 355 Japanese patients who enrolled in the study, 353 completed the study. The distributions of six representative Tag SNP genotypes are shown in Table 2. No observed genotype frequencies were significantly different from Hardy-Weinberg equilibrium.

**Table 2:** **Distribution of six Tag SNP genotypes examined in the** ***P2RX7*** **gene**

rs2708092	AA:	99	(28.0%)	/	AG:	177	(50.0%)	/	GG:	79	(22.0%)
rs1180012	CC:	128	(36.1%)	/	CT:	169	(47.6%)	/	TT:	58	(16.3%)
rs1718125	GG:	180	(50.7%)	/	GA:	149	(42.0%)	/	AA:	26	(7.3%)
rs208293	GG:	111	(31.3%)	/	GA:	177	(49.9%)	/	AA:	67	(18.9%)
rs1718136	AA:	247	(69.6%)	/	AG:	97	(27.3%)	/	GG:	11	(3.1%)
rs7132846	CC:	274	(77.2%)	/	CT:	75	(21.1%)	/	TT:	6	(1.7%)

The data are expressed as number (%) of subjects.

The Mann–Whitney *U*-test revealed that the VAS score at 24 h was significantly lower (*p* = 0.019; Table 3) in subjects who did not carry the minor A allele of the rs1718125 SNP than subjects who carried this allele, whereas the rs1718125 SNP had no significant association with PPLpre, PPLpost-PPLpre, 24-h postoperative fentanyl use, perioperative fentanyl use, total perioperative analgesic use, and the VAS score at 3 h. The unpaired *t*-test, Fisher’s exact test, and Mann–Whitney *U*-test revealed no significant differences in age, sex, duration of surgery, and duration of anesthesia between subjects who carried the minor A allele and subjects who did not carry this allele (*p* =0.079 [age: Table 3], *p* = 0.740 [sex: Table 3], *p* =0.430 [duration of surgery: AA + AG, 106 min (93, 128); GG, 105 min (92, 122)], and *p* = 0.384 [duration of anesthesia: AA + AG, 173 min (160, 195); GG, 173 min (157, 193)], respectively). Therefore, we did not conduct additional multivariate covariate analyses. The other five SNPs did not show any associations with any of the clinical endpoints (Table 3). When multiple-testing corrections (i.e., the standard Bonferroni correction for the number of SNPs) were applied, no significant association was found between the genotype of the rs1718125 SNP and VAS score at 24 h.

**Table 3:** **Patients’ demographic and clinical data**

	rs2708092		rs1180012		rs1718125	
	AA	AG + GG	CC	CT + TT	GG	GA + AA
Age (years)	26.1 ± 7.0	25.8 ± 7.8	27.3 ± 7.7	25.1 ± 7.5	26.6 ± 7.7	25.2 ± 7.5
Male/Female	32/67	92/163	51/77	74/153	65/115	60/115
PPLpre (s)	15 [10,21]	14 [9,23]	14 [9,23]	14 [9, 23]	14 [9, 22]	15 [9, 23]
PPLpost (s)	29 [18, 52]	27 [16, 54]	28 [16, 49]	28 [16, 54]	27 [16, 49]	30 [16, 56]
Analgesic effect (PPLpost-PPLpre) (s)	15 [5, 35]	11 [4, 35]	10 [4, 30]	13 [4, 36]	10 [4, 30]	14 [5, 37]
24-h postoperative fentanyl use (μg/kg)	2.2 [1.2, 4.7]	2.3 [1.0, 4.1]	2.3 [0.9, 4.1]	2.3 [1.1, 4.3]	2.1 [0.9, 3.9]	2.4 [1.3, 4.4]
Perioperative fentanyl use (μg/kg)	7.6 [5.8, 9.2]	6.7 [5.1, 9.1]	6.8 [5.0, 9.1]	7.1 [5.3, 9.1]	6.8 [5.0, 8.6]	7.1 [5.5, 9.5]
Total perioperative analgesic use (μg/kg)	8.5 [6.7, 10.2]	7.6 [6.0, 10.1]	7.7 [5.8, 10.2]	8.0 [6.2, 10.0]	7.8 [5.8, 9.6]	8.0 [6.3, 10.5]
VAS pain score at 3 h (mm)	27 [15, 47]	27 [15, 50]	27 [15, 50]	30 [15, 50]	27 [15, 50]	30 [16, 50]
VAS pain score at 24 h (mm)	25 [10, 38]	25 [10, 45]	22 [8, 40]	26 [11, 43]	23 [8, 40]	28 [14, 46]*
	**rs208293**		**rs1718136**		**rs7132846**	
	**GG**	**GA + AA**	**AA**	**AG + GG**	**CC**	**CT + TT**
Age (years)	27.5 ± 7.9	25.2 ± 7.4	26.2 ± 7.9	25.4 ± 6.9	25.7 ± 7.3	26.8 ± 8.8
Male/Female	41/70	84/160	95/152	30/78	104/170	21/60
PPLpre (s)	13 [9, 22]	15 [9, 24]	14 [9, 24]	15 [9, 21]	14 [9, 23]	14 [9, 23]
PPLpost (s)	27 [16, 51]	28 [16, 54]	29 [16, 56]	25 [15, 48]	28 [16, 53]	28 [16, 53]
Analgesic effect (PPLpost-PPLpre) (s)	10 [3, 34]	13 [5, 35]	13 [5, 36]	11 [4, 30]	12 [4, 35]	12 [4, 35]
24-h postoperative fentanyl use (μg/kg)	2.3 [1.1, 4.0]	2.3 [1.0, 4.3]	2.3 [1.1, 4.3]	2.0 [1.0, 4.1]	2.3 [1.1, 4.2]	2.3 [1.0, 4.2]
Perioperative fentanyl use (μg/kg)	6.8 [5.1, 9.0]	7.1 [5.3, 9.2]	7.0 [5.2, 9.2]	6.7 [5.2, 8.9]	6.9 [5.2, 9.1]	6.9 [5.2, 9.1]
Total perioperative analgesic use (μg/kg)	7.7 [5.9, 10.1]	8.0 [6.2, 10.1]	8.0 [6.1, 10.1]	7.7 [6.1, 9.9]	8.0 [6.1, 10.1]	8.0 [6.1, 10.1]
VAS pain score at 3 h (mm)	27 [16, 50]	27 [15, 49]	27 [15, 50]	26 [15, 49]	28 [15, 50]	27 [15, 50]
VAS pain score at 24 h (mm)	22 [10, 39]	26 [11, 45]	25 [10, 42]	25 [8, 42]	25 [10, 42]	25 [10, 42]

The data are expressed as numbers, mean ± SD (range), or median [interquartile range]. *p <0.05, compared with subjects not carrying minor allele.

We further analyzed the haplotype-based associations of the six *P2RX7* gene Tag SNPs with clinical endpoints. Of the 26 estimated haplotypes, 10 haplotypes (estimated frequency >1%) are listed in Table 4. Haplotype No.1 (ACGGAC) was significantly associated with both higher PPLpre score and higher PPLpost-PPLpre score in the linear regression analysis that used the recessive model (Table 5) and log-additive model (PPLpre: coefficient = 4.532 [confidence interval (CI): 0.048, 9.017], z-score = 1.981, *p* =0.048; PPLpost-PPLpre: coefficient = 8.735 [CI: 1.829, 15.640], z-score = 2.479, *p* = 0.013) and associated with higher PPLpost-PPLpre score using the dominant model (coefficient =8.802 [CI: 0.601, 17.004], z-score = 2.104, *p* = 0.035), although the adjusted *p* values of these associations with haplotype No.1 after multiple-testing corrections (i.e., the standard Bonferroni correction for the number of clinical endpoints) were not significant. The linear regression analyses that used the recessive model revealed that haplotype No.3 (GTAAAC) was significantly associated with both lower PPLpre score and lower PPLpost-PPLpre score, even after multiple-testing corrections (Table 5). These significant associations were not evident when analyzed using either a dominant or log-additive model. Furthermore, linear regression analyses that used the recessive model also revealed that haplotype No.2 (GCGGAC) was significantly associated with lower VAS scores at 24 h, even after multiple-testing corrections (Table 5). This significant association was also not evident when analyzed using either the dominant or log-additive model.

**Table 4:** **Estimated** ***P2RX7*** **gene Tag SNP haplotypes and their frequencies**

	Estimated haplotypes	Frequency	(SE)
1	ACGGAC	0.245	(0.016)
2	GCGGAC	0.229	(0.019)
3	GTAAAC	0.150	(0.014)
4	ATGAGC	0.103	(0.011)
5	GTAAAT	0.051	(0.010)
6	ACGGAT	0.048	(0.011)
7	ATAAGC	0.046	(0.009)
8	ACGAAC	0.041	(0.008)
9	ATAAAC	0.018	(0.014)
10	GTGGAC	0.016	(0.006)

**Table 5:** **Associations between** ***P2RX7*** **gene Tag SNP haplotypes and clinical data**

		PPLpre				
	Haplotypes	Coefficient	SE	CI	z-score	***p***value
1	ACGGAC	13.245	6.576	(0.357, 26.134)	2.014	0.044
2	GCGGAC	-0.23	4.252	(-8.563, 8.103)	-0.05	0.957
3	GTAAAC	-7.275	2.34	(-11.860, -2.689)	-3.11	0.002
		**Analgesic effect (PPLpost-PPLpre)**				
		**Coefficient**	**SE**	**CI**	**z-score**	***p*** **value**
1	ACGGAC	19.452	9.34	(1.146, 37.759)	2.083	0.037
2	GCGGAC	-2.966	6.87	(-16.431, 10.499)	-0.43	0.666
3	GTAAAC	-14.97	4.585	(-23.957, -5.983)	-3.27	0.001
		**VAS pain score at 24 h**				
		**Coefficient**	**SE**	**CI**	**z-score**	***p*** **value**
1	ACGGAC	7.878	4.067	(-0.094, 15.849)	1.937	0.053
2	GCGGAC	-10.799	3.523	(-17.705, -3.894)	-3.07	0.002
3	GTAAAC	7.434	8.88	(-9.971, 24.839)	0.837	0.402

Haplotype-based associations were tested using linear regression analyses with a recessive model.

## Discussion

We studied patients who underwent mandibular sagittal split ramus osteotomy. Subjects who undergo this cosmetic surgery are usually young and healthy. The operation causes considerable perioperative pain that arises from the dissected mandibular bone, and the surgical technique is highly standardized at our institute. We conducted a standardized pain test before the induction of general anesthesia in opioid-naive subjects without pain. Using these ideal subjects and methods, we found that PPLpre and the analgesic effects of fentanyl (PPLpost-PPLpre) evaluated in the cold pressor test were significantly lower in subjects who carried homozygous haplotype No.3 (GTAAAC) in the *P2RX7* gene compared with the other subjects. Subjects who carried haplotype No.1 (ACGGAC), which has a different allele in the Tag SNP on the LD1-3 regions from haplotype No.3, tended to have higher PPLpre scores and higher analgesic effects of fentanyl. These results suggest that the combination of polymorphisms from the 5′-flanking region to exon 5 (from LD1 to LD3 regions) in the *P2RX7* gene could recessively affect cold pain sensitivity and the analgesic effects of opioids for acute cold pain. Furthermore, VAS scores at 24 h tended to be lower in subjects who carried homozygously the major G allele of the rs1718125 SNP (3rd Tag SNP) than subjects who carried the minor A allele. We also found that VAS scores at 24 h were significantly lower in subjects who carried homozygous haplotype No.2 (GCGGAC) in the *P2RX7* gene compared with the other subjects. Thus, haplotype No.2 could recessively affect the postoperative analgesic effects of opioids. Although we analyzed five types of BeadChips that were merged together in the present study because the sample sizes were small for each of the five datasets and they all presumably had Japanese ancestry, performing a meta-analysis is usually better than merging. Thus, further studies that have a greater number of samples might be required to reveal the influences of these haplotypic effects in the *P2RX7* gene. Twenty-four hour postoperative fentanyl use, perioperative fentanyl use, and total perioperative analgesic use were not associated with haplotypes in the *P2RX7* gene. Although further validation is needed, the analgesic/opioid requirements for postoperative pain management might not be associated with genetic polymorphisms in the *P2RX7* gene.

In the present study, subjects who carried homozygous haplotype No.3 (GTAAAC) in the *P2RX7* gene exhibited higher cold pain sensitivity and lower analgesic effects of fentanyl for acute cold pain in the cold pressor test, but still unknown is whether this combination of SNPs alters gene function or expression, which may be an important limitation of the present study. Many SNPs have been identified in the *P2RX7* gene, and some of the SNPs in the *P2RX7* gene have been shown to cause changes in receptor function [22–24]. Although our present study did not focus on these reported functional SNPs, some of them are located on the present LD blocks. The rs208294 T/C SNP (Tyr^155^ to His), which was described as the C489T SNP in previous reports with non-Japanese subjects, is located in the LD3 region in the present study. This SNP showed high LD (*D’* =1.0) with the present 4th Tag SNP, rs208293 G/A SNP. In the previous report, functional analyses in recombinant cells that expressed P2X_7_ receptors with the mutation of the rs208294 SNP using [Ca^2+^]_i_ influx and ethidium uptake experiments revealed that the T > C mutation in this position caused a loss of function of P2X_7_ receptors [34]. Although the rs208293 G/A SNP alone did not show any association with cold pain sensitivity and the analgesic effects of fentanyl, the rs208294 T/C SNP might be one of the mutations in LD1-3 that caused the present results. Interestingly, P2X_7_ receptors have been reported to contain several alternative splicing variants (P2X7_a-k_), some of which presented altered function [35–38]. Because the splicing difference in more than half of these isoforms occurs from the LD1 to LD3 regions (from exon 1 to exon 5) in the *P2RX7* gene, polymorphisms in these regions might affect this splicing mechanism and induce a functional change in neuronal transmission via P2X_7_ receptors. Further studies that focus on gene polymorphisms from LD1 to LD3 in the *P2RX7* gene may reveal the functional mechanisms that affect pain sensitivity and clinical efficacy of opioids. A recent report showed a genetic association between the hypofunctional rs7958311 G/A SNP (Arg^270^ to His) and lower pain intensity in two cohort studies of human patients with chronic pain [24]. The rs7958311 G/A SNP is located in the gap between the present LD4 and LD5 regions. Including this SNP, some other nonsynonymous SNPs (e.g., rs3751143 A/C SNP [Glu^496^ to Ala] [22]) are located in the gap among the present LD regions and were not evaluated in the present study. Further association analyses of these SNPs in the *P2RX7* gene may be necessary to reveal the role and mechanisms of P2X_7_ receptors in naive pain sensitivity and the efficacy of analgesics.

The potential of P2X_7_ receptors as a therapeutic target in the management of neuropathic pain and inflammation has been the subject of intensive recent investigations [2, 3, 39], but the involvement of P2X_7_ receptors in the modulation of naive pain sensitivity and efficacy of analgesics is still unclear. P2X_7_ receptors are expressed predominantly on immune cells, and are speculated to contribute to the hyperexcitability of nociceptive neurons through the release of both interleukin-1β (IL-1β) and tumor necrosis factor α (TNF-α) [40–42]. These cytokines are well known to play important roles in the generation and maintenance of pain, and especially in chronic pain states [39, 42]. P2X_7_ receptors are also known to act not only as cation channels following brief activation by extracellular ATP but also as large nonselective pores following prolonged or repeated activation. Sorge et al. reported that mice that carried a mutation in the *P2RX7* gene that causes impaired pore formation showed less hypersensitivity in neuropathic and inflammatory pain states [24]. These previous data might indicate that the pore formation of P2X_7_ receptors closely affects chronic pain states [43]. In the present study, haplotype No.2 (GCGGAC) and the rs1718125 SNP in the *P2RX7* gene showed associations with VAS scores at 24 h but not with VAS scores at 3 h or the analgesic effects of fentanyl in the cold pressor test. Immediate pain transmission in the cold pressor test and the acute phase of postoperative pain (reflected by VAS score at 3 h) might have different pain-modulated mechanisms from subacute postoperative pain (VAS score at 24 h), and the latter might be affected by the pore formation of P2X_7_ receptors. Further validation of the relationship between these *P2RX7* gene polymorphisms and time-dependent changes in postoperative pain states is needed to clarify this issue.

The present study revealed that PPLpre and PPLpost-PPLpre in the cold pressor test were associated with haplotypes of the SNPs (haplotype No.1 and No.3) in the *P2RX7* gene, suggesting that P2X_7_ receptors are involved in cold pain transmission and the analgesic effects of opioids for cold pain stimuli under naive conditions. However, these haplotypes showed no significant association with clinical pain conditions (VAS scores) or fentanyl requirements. The pain stimulus in the cold pressor test may be mediated by the activation of transient receptor potential ankyrin 1 (TRPA1) channels in primary sensory neurons [44]. Postoperative pain may be mediated mainly by the activation of transient receptor potential vanilloid 1 (TRPV1) channels subsequent to incision and inflammation and not by the activation of TRPA1 channels [45]. P2X_7_ receptors are well known to act as ATP-gated Ca^2+^-permeable cation channels. Furthermore, the function of TRP channels might be regulated by external Ca^2+^ [46]. Thus, together with our present results, TRPA1 channels may be affected by the channel function of P2X_7_ receptors, and TRPV1 channels may be affected by the pore formation of P2X_7_ receptors. Although further investigation and validation are needed to confirm this hypothesis, P2X_7_ receptors are suggested to play differential roles in pain transmission, depending on the type of pain stimulus. Furthermore, the mechanisms of P2X_7_ receptor-induced alterations in the analgesic effects of opioids are still unclear. Only a few reports have shown that the blockade of P2X_7_ receptors with specific antagonists or targeting small interfering RNA (siRNA) enhanced the analgesic effects of morphine in chronic morphine-treated (i.e., morphine-tolerant) rats, although these effects were not evident in naive rats [47, 48]. Thus, neuronal modulation via P2X_7_ receptors may affect the analgesic effects of opioids, but these modulatory effects may depend on the type of pain stimulus or pain expression (i.e., acute or chronic).

## Conclusions

In Japanese subjects, naive cold pain sensitivity and the analgesic effect of fentanyl were related to an SNP and haplotypes in the *P2RX7* gene. The rs1718125 G > A SNP tended to be associated with higher VAS scores at 24 h after mandibular sagittal split ramus osteotomy. Furthermore, subjects with homozygous haplotype No.3 (GTAAAC) exhibited higher cold pain sensitivity and lower analgesic effects of fentanyl for acute cold pain in the cold pressor test. The combination of polymorphisms from the 5′-flanking region to exon 5 in the *P2RX7* gene could recessively affect cold pain sensitivity and the analgesic effects of opioids for acute cold pain. Subjects with homozygous haplotype No.2 (GCGGAC) had lower VAS scores at 24 h after surgery. Although further validation is needed, our data may provide valuable information about the role of P2X_7_ receptors in pain pathways and pain treatment.

## Electronic supplementary material

Additional file 1: **State of linkage disequilibrium (LD) between the SNPs in the** ***P2RX7*** **gene.** The numbers in squares represent percentages of the *D’* values. Squares without numbers represent *D’* = 1. The color scheme is presented according to the “Standard Color Scheme” of Haploview v.4.2 software: bright red (Lod ≥ 2, *D’* = 1), shades of pink/red (Lod ≥ 2, *D’* < 1), blue (Lod < 2, *D’* = 1), white (Lod < 2, *D’* < 1). Blue lines indicate the exon regions of the *P2RX7* gene. (PPT 338 KB)
