# Supplementary figures and images for: Haplotypes of P2RX7 gene polymorphisms are associated with both cold pain sensitivity and analgesic effect of fentanyl
Source: Mol Pain. 2014 Dec 3;10:75. doi: 10.1186/1744-8069-10-75 (PMC4265416; doi:10.1186/1744-8069-10-75)

## Slide 1
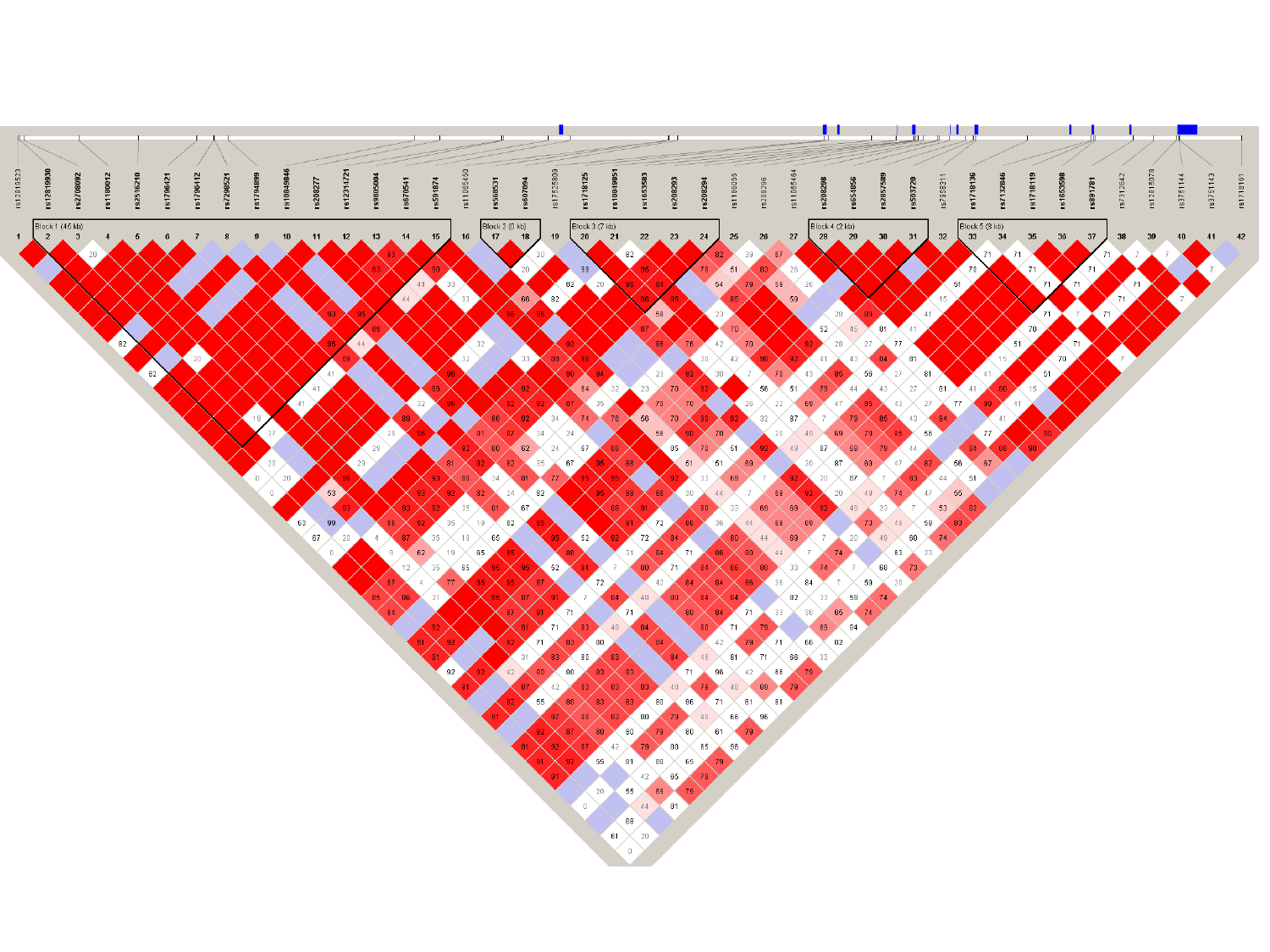

Supplement: Supplementary file 1 — Additional file 1: State of linkage disequilibrium (LD) between the SNPs in the P2RX7 gene. The numbers in squares represent percentages of the D’ values. Squares without numbers represent D’ = 1. The color scheme is presented according to the “Standard Color Scheme” of Haploview v.4.2 software: bright red (Lod ≥ 2, D’ = 1), shades of pink/red (Lod ≥ 2, D’ < 1), blue (Lod < 2, D’ = 1), white (Lod < 2, D’ < 1). Blue lines indicate the exon regions of the P2RX7 gene. (PPT 338 KB) [file 12990_2014_678_MOESM1_ESM.ppt]
